# Supplementary figures and images for: Antibiotic prophylaxis and hospitalization of horses subjected to median laparotomy: gut microbiota trajectories and abundance increase of Escherichia
Source: Front Microbiol. 2023 Nov 23;14:1228845. doi: 10.3389/fmicb.2023.1228845 (PMC10701544; doi:10.3389/fmicb.2023.1228845)

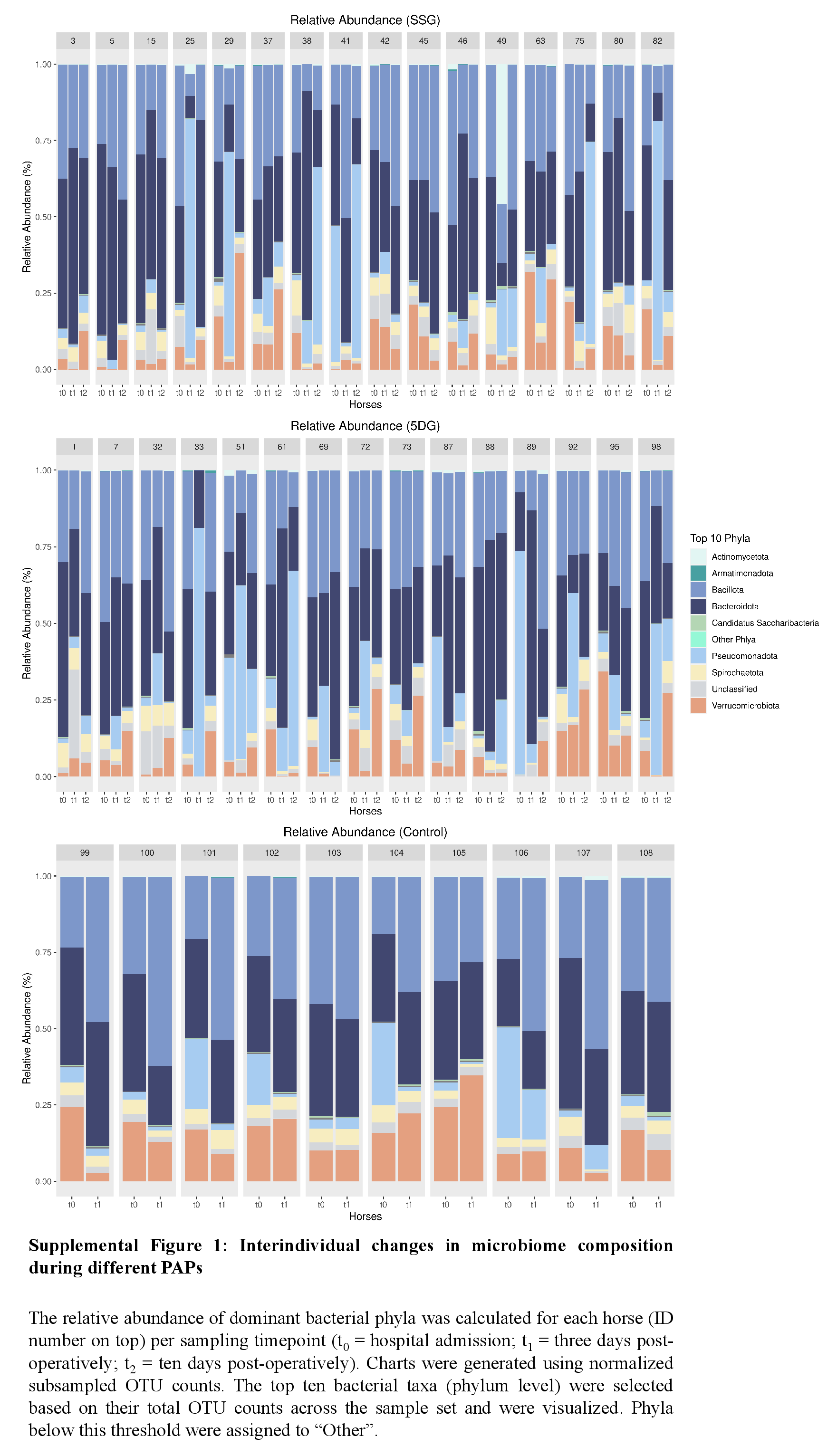

Supplement: Supplementary file 2 [file Image_1.TIFF]
